# Hemodynamic evaluation of endarterectomy and stenting treatments for carotid web

**DOI:** 10.3389/fcvm.2022.993037

**Published:** 2022-10-20

**Authors:** Shuqi Ren, Qijia Liu, Zengsheng Chen, Xiaoyan Deng, Anqiang Sun, Jingyuan Luan

**Affiliations:** ^1^Key Laboratory for Biomechanics and Mechanobiology of Ministry of Education, Beijing Advanced Innovation Center for Biomedical Engineering, School of Biological Science and Medical Engineering, Beihang University, Beijing, China; ^2^Interventional Radiology and Vascular Surgery, Peking University Third Hospital, Beijing, China

**Keywords:** carotid web, numerical simulation, hemodynamics, stent, stroke

## Abstract

**Background and purpose:**

A carotid web is a thin, shelf-like luminal protrusion in the internal carotid artery that might cause carotid stenosis and stroke by inducing disturbed flow patterns, thrombosis, and abnormal biomechanical stimulus to the endothelial cells. This study simulated and evaluated how the two main treatments (endarterectomy and stenting) influence hemodynamic environments in the carotid artery and distal carotid siphon arteries, aiming to provide more references for the selection of clinical treatment.

**Materials and methods:**

The carotid web, endarterectomy, and stenting models were reconstructed based on CT images. The blood flow simulations were conducted, and critical parameters related to thrombosis formation and artery remodeling, including swirling strength, wall shear stress (WSS), vortex Q-criterion, and oscillating shear index (OSI), were analyzed.

**Results:**

In the model of the carotid web, obvious recirculation formed distal to the web, accompanied by lower velocity, lower WSS, higher relative resident time (RRT), and higher Q value. While in both two treatment models, the velocity increased and the Q value and RRT decreased at the carotid bifurcation. In addition, both treatments provide more kinetic energy to the distal carotid siphon artery, especially the stenting model.

**Conclusion:**

The carotid web can significantly influence the flow environments in the carotid artery. Both endarterectomy and stenting treatments could significantly diminish the side effects of the web and are feasible choices for web patients in terms of hemodynamics. Besides, the treatments for the carotid web would also influence the flow patterns at the distal carotid siphon, especially for the stenting treatment. But more innovational designs are needed to make the minimally invasive stenting treatment more beneficial.

## Introduction

A carotid web is a thin, shelf-like luminal protrusion of the intimal fibrous tissue in ICA, causing the narrowing of the carotid bulb as a non-atherosclerotic disease of the extracranial carotid vasculature (1). It was initially reported as a filling defect in 1968 and then gradually depicted as a web-like septum (2). The prevalence of the carotid web was reported to be ~1.2% (3). Although the carotid web rarely happens, it is getting more and more recognized as a cause of acute ischemic stroke, particularly in young female adults (4, 5).

For carotid artery stenosis caused by atherosclerotic plaques, carotid endarterectomy (CEA) and carotid artery stenting (CAS) are both routinely performed in clinical practice, both of which have been shown to significantly reduce the risk of strokes. In addition, it is generally accepted that CEA is preferred over CAS unless general anesthesia is contraindicated or frailty (6, 7). Tulamo et al. suggested that CAS was associated with a slightly greater risk of death and stroke than CEA in patients with asymptomatic carotid stenosis (8). Previous numerical simulation studies have investigated the hemodynamic characteristics and the possibility of restenosis after CEA for carotid stenosis caused by atherosclerotic plaques. Kamenskiy et al. investigated the biomechanics of different patch widths and locations of closure during carotid angioplasty, providing a reference for the selection of patches (9). Guerciotti et al. provided the computational study of the effects of CEA on the fluid dynamics at internal carotid bifurcations, and the results have shown that CEA can restore physiological fluid dynamic conditions, though the risk of restenosis was increased (10).

At present, the treatments for carotid web also mainly include CEA and CAS (5). Brinjikji et al. (2, 4) suggested that stenting treatment might be performed feasibly. Joux et al. (11) showed that surgical removal could be a therapeutic option to limit recurrent strokes. There is no evidence yet of which treatment option is the most preferred.

The web would certainly change the carotid geometry and obstruct the physiological blood flow downstream of the internal carotid. K. C. J. Compagne and colleagues' pioneering study has investigated the hemodynamic feature of the carotid web and stated that a carotid web might stimulate thrombosis formation by disturbing the blood flow (12). It has been found that thrombosis and arterial pathology are strongly correlated with hemodynamic characteristics in the artery (13). The formation and accumulation of intraluminal thrombosis are usually related to disturbed flow, representing low WSS, high RRT, and high vortex (14, 15). Therefore, it is also essential to evaluate and compare the hemodynamic changes after the two treatments.

The purpose of this study was to numerically simulate the flow patterns in the carotid models after the endarterectomy and stenting treatments, respectively, and compare them with the situation in the web model before treatment, aiming to provide more understanding of the web and more references for the selection of clinical treatment.

## Materials and methods

### Models

**Figure 1:**
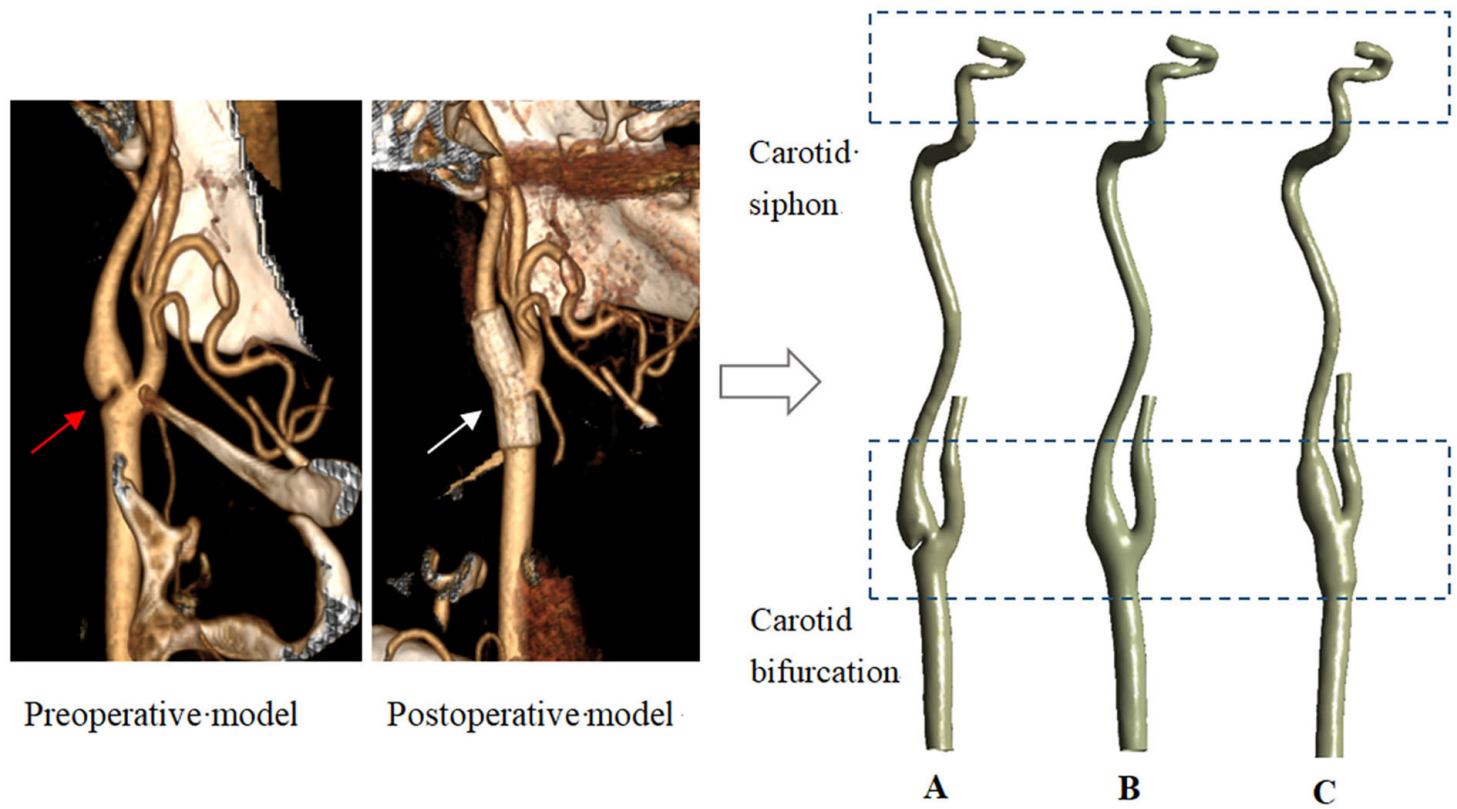
Three models reconstructed from CT data. (**A**–carotid webs model; **B**–endarterectomy model; **C**—stenting model. The red and white arrows point to the web and the stent).

In this study, three models were used for hemodynamic simulation, namely, the carotid model with web (Model A), the endarterectomy model (Model B), and the stenting model (Model C). Model A and Model C were reconstructed based on CT images of a carotid web patient scanned before and after stenting treatment. According to the clinical case of Shen et al., for the web specimen, histopathologic examination showed fibrocytes, mainly elastic fibers, with no evidence of atherosclerosis (16). In addition, the study has shown that the primary closure of CEA has a minimal effect on preoperative geometry (17), so Model B was reconstructed based on Model A, only in the absence of the web part. All the CT images were scanned at the Third Hospital at Peking University. The patient-relevant parameters of CT data were as follows: the slice thickness was 0.625 mm, the reconstruction spacing/increment was 0.625 mm, and the image resolution was 512 × 512. The Mimics software (version 15.0, Materialize, Ann Arbor, MI, USA) was used to reconstruct 3D models using these images. Light smoothing of these models was applied using Geomagic Studio (version 12.0, Geomagic, USA). To evaluate the influence of treatments on the distal arteries, all three models are built from the common carotid to the carotid siphon part. The carotid web, endarterectomy, and stenting treatment models are shown in Figure 1. In addition, to quantitatively evaluate the effects of different treatments on the siphon area, eight cross sections were established (as shown on the left of **Figure 9**).

### Mesh generation

The computational meshes created using ANSYS ICEM CFD (ANSYS Inc., Canonsburg, PA) were all hexahedral and tetrahedral dense grids. Grids of three densities were simulated under the same conditions (Model A: 838578, 1095195, and 2523077; Model B: 807021, 1097561, and 2515025; Model C: 1094428, 1455268, and 3353938), and the area-averaged velocity of eight cross sections was utilized to assess the grid independence (the relative error was < 5%). To save computing resources on the premise of ensuring the calculation accuracy, the final element numbers of Models A, B, and C were 838578, 807021, and 1094428, respectively.

### Boundary conditions

**Figure 2:**
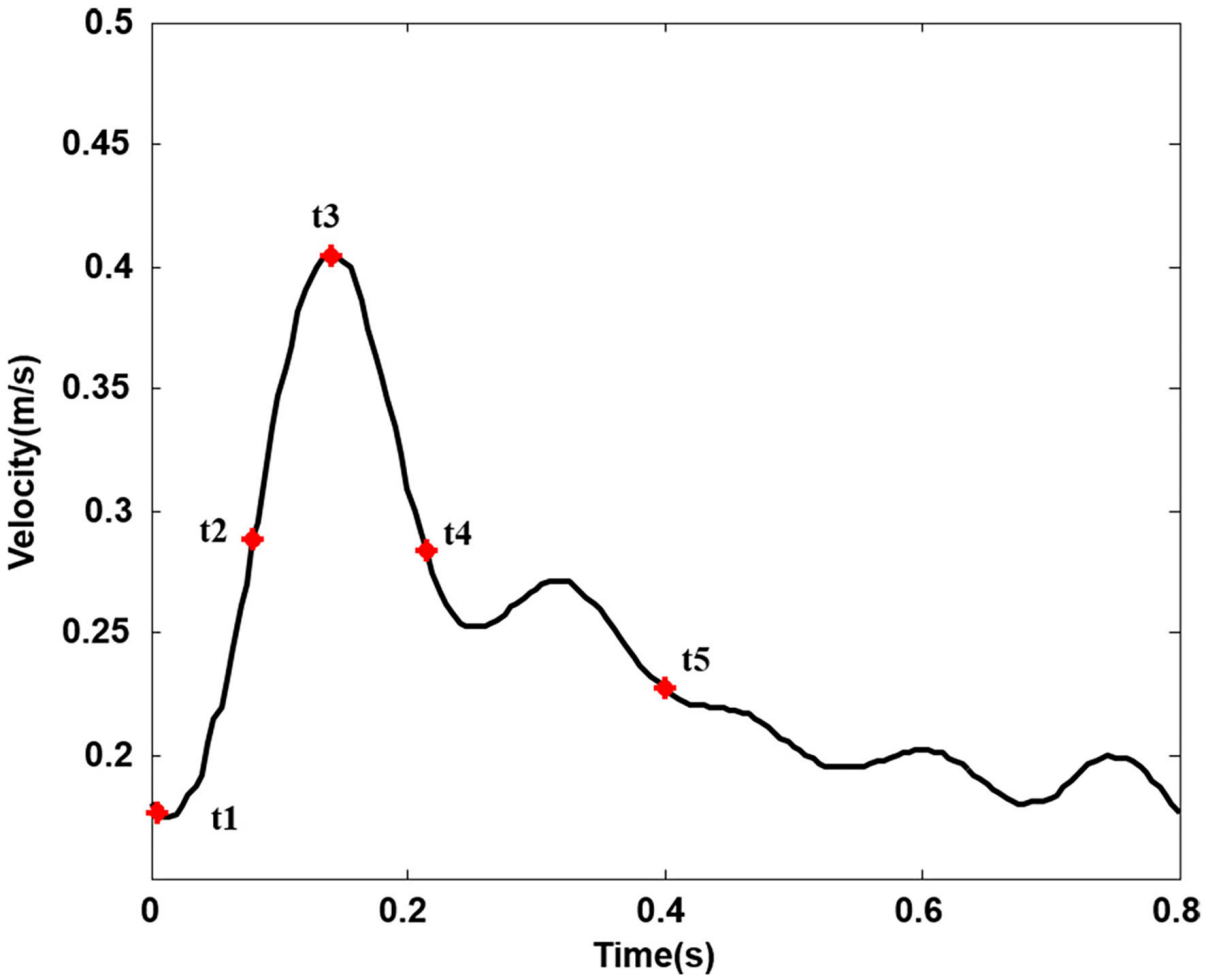
Inlet velocity pulse in the common carotid artery. (Five typical points during one cardiac cycle. t1, t2, t3, t4, and t5, respectively, represent start systole, early systole, peak systole, late systole, and early diastole).

The numerical study was computed under pulsatile flow conditions according to previous studies (12). The physiological inlet flow wave is shown in Figure 2 (18). The outlet has been extended accordingly to avoid the influence of boundary conditions on flow to a certain extent, and the outflow ratio of internal and external carotid arteries was 0.65:0.35 (10, 19). All the artery walls and stents were assumed to be rigid and nonslip, which has been considered to be reasonable in lots of peripheral artery studies (20, 21).

### Governing equations and iterations

The blood was assumed to be an incompressible, homogenous, and Newtonian fluid. The numerical simulation for flow motion was based on the three-dimensional incompressible Navier-Stokes equations and continuity equation:


$$\begin{array}{l} \rho \left[\frac{\partial \vec{\text{u}}}{\partial \text{t}}+\left(\vec{\text{u}}\cdot \nabla \right)\vec{\text{u}}\right]+\nabla p-\mu \cdot \Delta \vec{\text{u}} \end{array}$$

$$\begin{array}{l} \nabla \cdot \vec{\text{u}}=0 \end{array}$$

where $\vec{u}$ and p represent, respectively, the fluid velocity vector and the pressure, ρ = 1,050 kg/m^3^ and μ = 3.5 × 10^−3^ kg/m· s are the density and dynamic viscosity of blood (22).

Simulations were performed using the finite volume method. During the calculation, discretization of the pressure and momentum at each control volume was in a second-order upwind. The time step is set to 0.005; after four cycles, the results tend to be stable, and the continuity and velocity residuals converge to 1.0 × 10^−5^.

### Hemodynamic quantities of interest

#### The Q-criterion definition of a vortex

The Q-criterion was used to quantitatively describe vortices in the flow field. This method has been used to study the formation and evolution of vortex structures, which has verified the feasibility and potential value of vortex visualization (23). The vortex zones can be identified by the Q-criterion when rotational flow dominates over straining flow; that is to say, the antisymmetric vorticity tensor ($\vec{\vec{\Omega }}$) part of the vortex zone is greater than the symmetric strain rate tensor ($\vec{\vec{S}}$) part (24, 25):


$$\begin{array}{l} \text{Q}=\frac{1}{2}\left[\|\vec{\vec{\Omega }}{\|}^{2}-\|\vec{\vec{\text{S}}}{\|}^{2}\right]>0 \end{array}$$

The mathematical expressions of the antisymmetric part, $\vec{\vec{\Omega }}$, and the symmetric part, $\vec{\vec{S}}$, are as follows:


$$\begin{array}{l} \vec{\vec{\Omega }}=\frac{1}{2}\left(\nabla \vec{\text{v}}-\nabla {\vec{\text{v}}}^{\text{T}}\right) \end{array}$$

and


$$\begin{array}{l} \vec{\vec{\text{S}}}=\frac{1}{2}\left(\nabla \vec{\text{v}}+\nabla {\vec{\text{v}}}^{\text{T}}\right) \end{array}$$

where formulas are defined by the gradient of the velocity field $\nabla \vec{v}$ and its transpose, respectively.

As can be seen from the above equations, when the anti-symmetric vorticity tensor part is dominant, Q > 0. Therefore, in this study, we define a vortex zone as a rotationally dominated flow domain with Q > 0 (26).

#### Swirling strength

Swirling strength can eliminate the influence of shear stress, and the vortex structure that cannot be identified by vorticity can be extracted by analyzing the velocity field so that the vortex structure in the flow field can be highlighted. According to Adrian et al. (27), the swirling strength method can be used as a reliable method to identify vortices and statistical vortices in turbulent field analysis.

#### WSS-based hemodynamic parameters

Based on the changes in wall shear stress during an entire cardiac cycle, we extracted three hemodynamic parameters, including the time-averaged wall shear stress (TAWSS), OSI, and RRT (28). Definitions of the WSS-based indicators are described as follows (29):


(1) $$\begin{array}{l} \text{TAWSS}=\frac{1}{\text{T}}\int_{0}^{\text{T}}|\text{WSS}\left(\text{s,t}\right)|\cdot \text{dt} \end{array}$$

where T is the lasting time of a pulsatile, and s is the position on the vessel wall.


(2) $$\begin{array}{l} \text{OSI}=\frac{1}{2}\left[1-\frac{\left|\int_{0}^{\text{T}}\text{WSSdt}\right|}{\int_{0}^{\text{T}}|\text{WSS}|\text{dt}}\right] \end{array}$$

The OSI is an index to describe the change frequency of WSS during a pulsatile cycle (30), indicating how even in time the WSS is positive and negative. It ranges from 0 to 0.5, where a value of 0.5 indicates a fully oscillatory shear case and a 0 value of OSI corresponds to unidirectional shear flow.


(3) $$\begin{array}{l} \text{RRT}=\frac{1}{\left(1-2\cdot \text{OSI}\right)\cdot \text{TAWSS}} \end{array}$$

The RRT is a parameter of the shear environment based on TAWSS and OSI, and it indicates the residence time of particles near the vessel wall.

## Results

**Figure 3:**
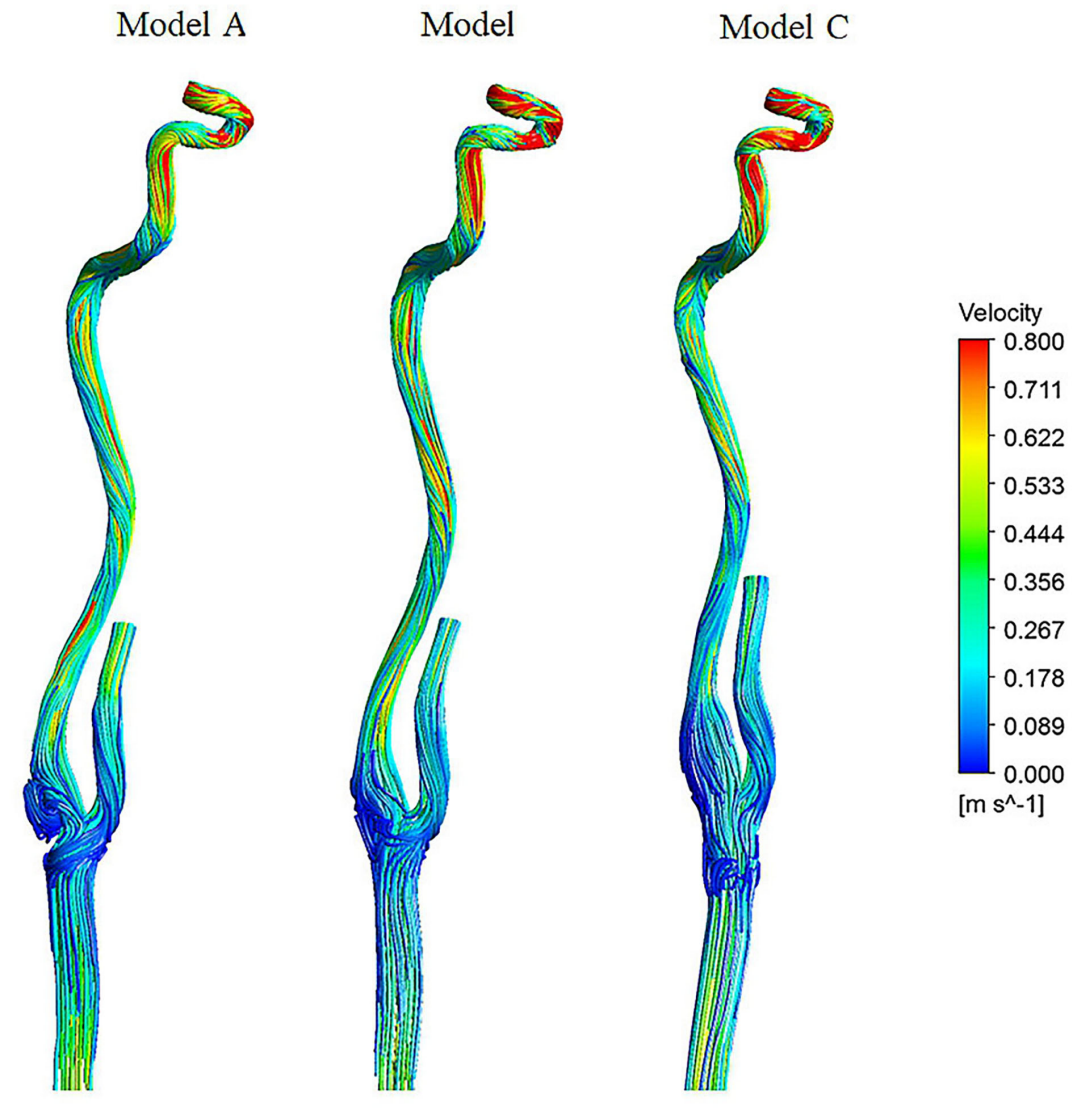
Streamlines colored by the magnitude of velocity at peak systole.

Figure 3 shows three-dimensional streamlines in three models at the peak systole. Flow patterns, recirculation areas, and high and low-velocity locations are shown in the three models. The following hemodynamic comparison mainly focused on the carotid bifurcation part and the siphon part.

### The hemodynamics characteristics at the carotid bifurcation

#### Flow pattern

**Figure 4:**
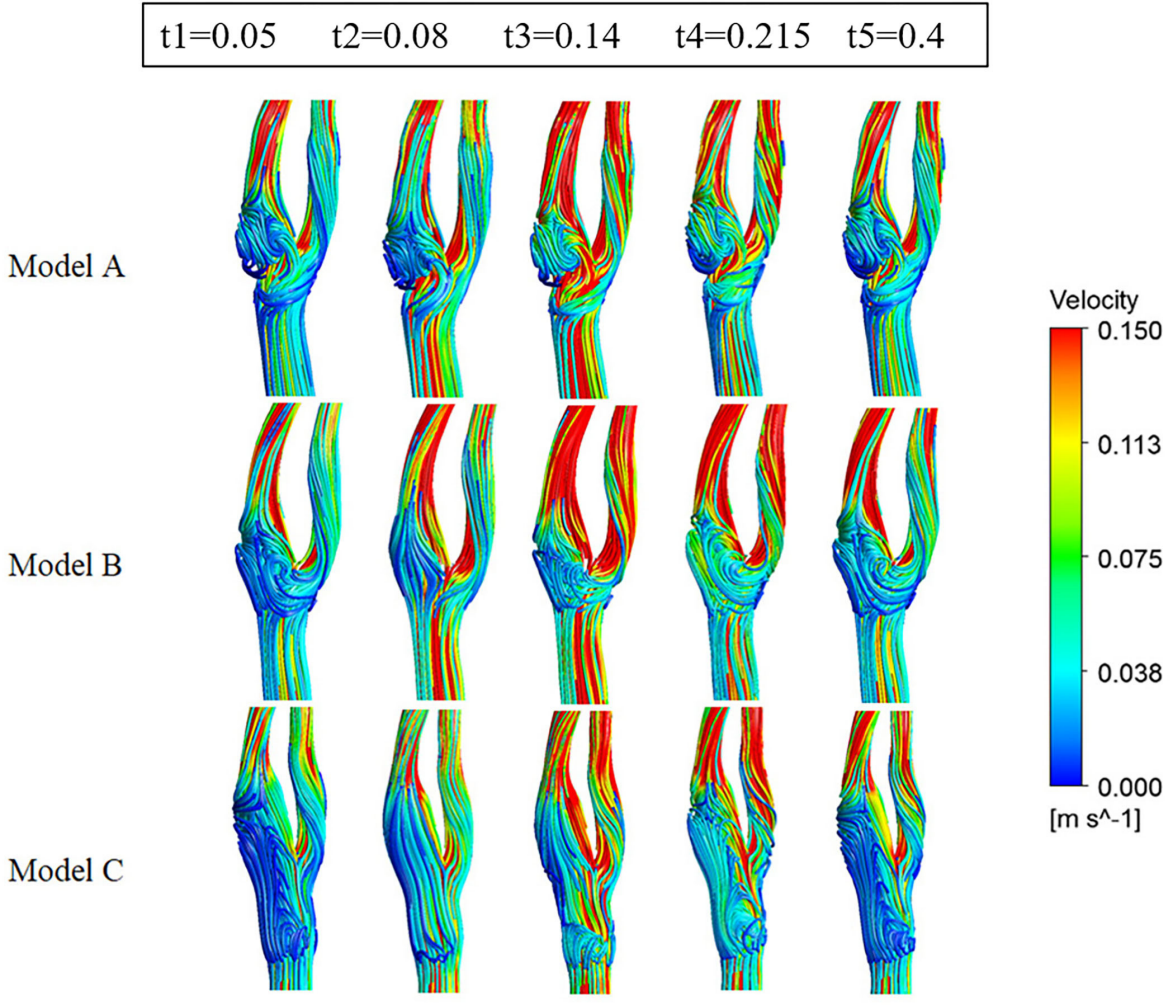
Streamlines colored by the magnitude of velocity at five time points.

**Figure 5:**
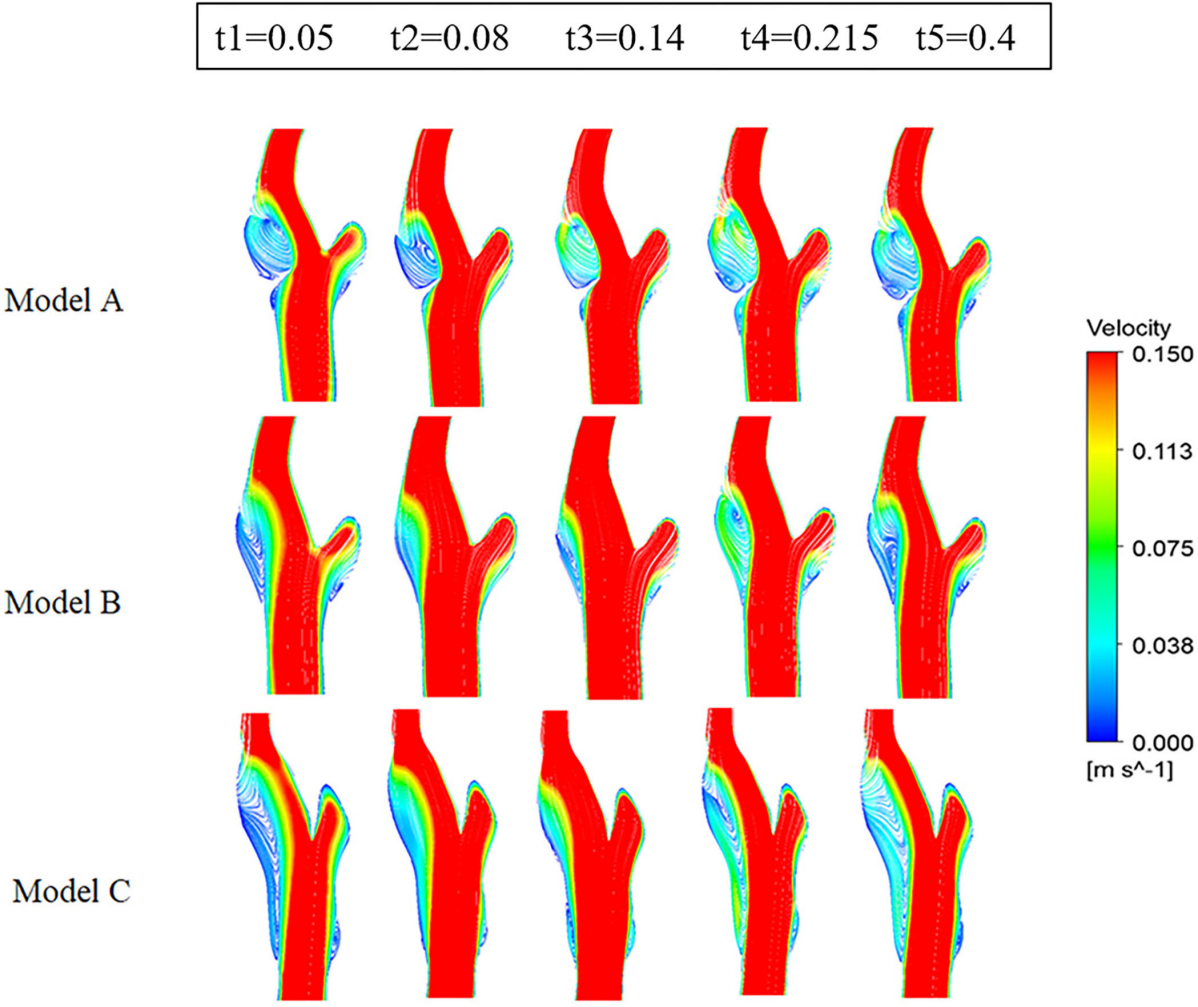
Velocity streamlines on cross sections at five points.

As shown in Figure 4 (3D stream lines) and Figure 5 (2D stream lines in the cross-section), each model has a low-speed recirculation zone near the outer wall of the internal carotid. But the geometries of the recirculation area are markedly different, with the widest recirculation for the web model (Model A) and the longest recirculation for the stenting model (Model C). In Model A, the recirculation zone distal to the web invaded more than half of the internal lumen, with obvious backflow and secondary vortex. Although the recirculation zone became narrow in Model C, it has been prolonged along the outer wall of the internal carotid with the same length as the stent.

**Figure 6:**
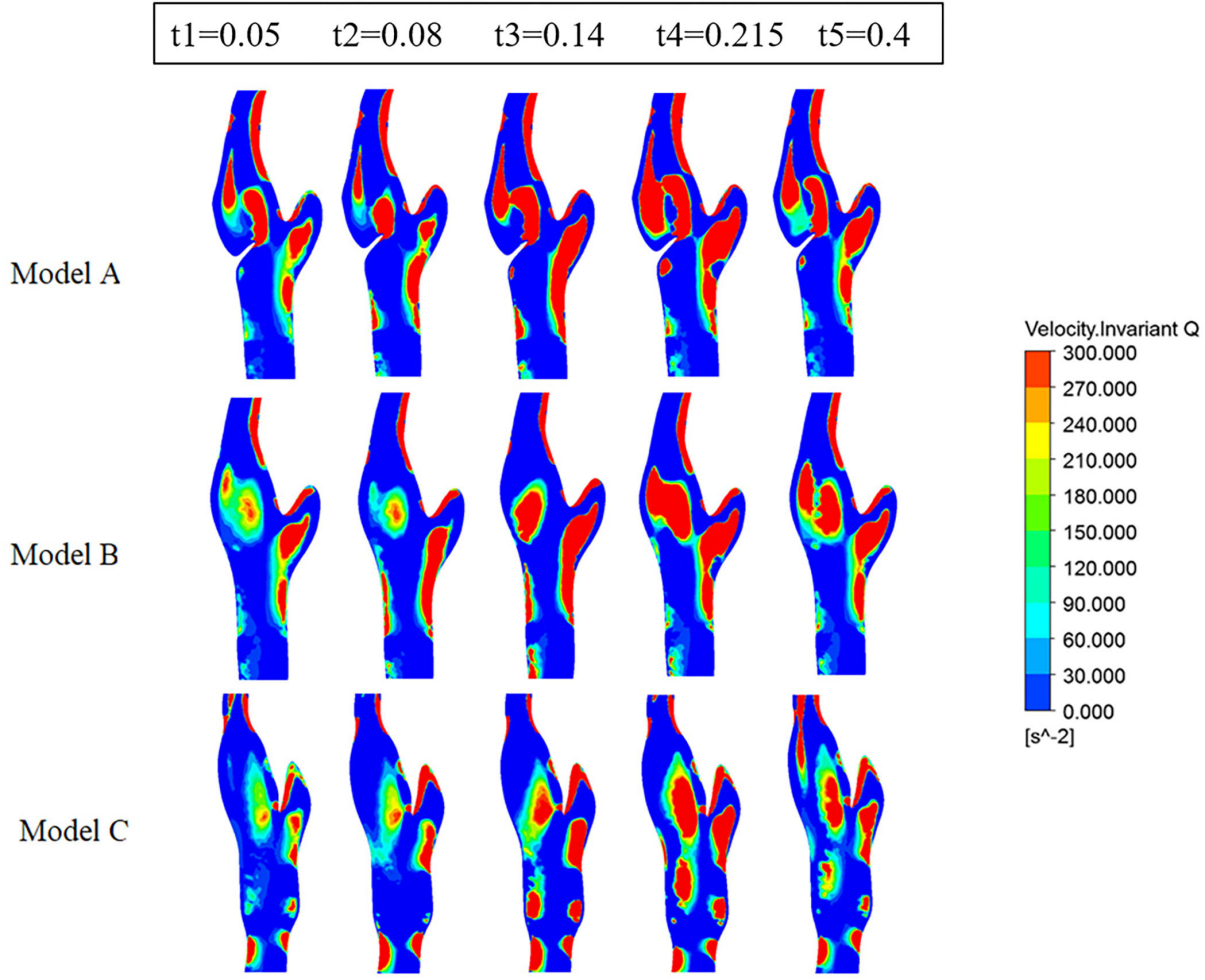
Contours of Q-criterion on the cross sections at five points.

Figure 6 represents the Q-value distribution on the cross sections, which can quantitatively describe the flow vortex in the flow field. High Q zones existed in all three models and along all the heart cycles, due to the bifurcation geometry of the carotid artery. The high Q zones are larger in Model A than in Model B and Model C, especially at the early systolic time points (t1, t2). When comparing Model B and Model C, the high Q zones in Model C are smaller and more dispersed.

#### WSS, OSI, and RRT distributions

**Figure 7:**
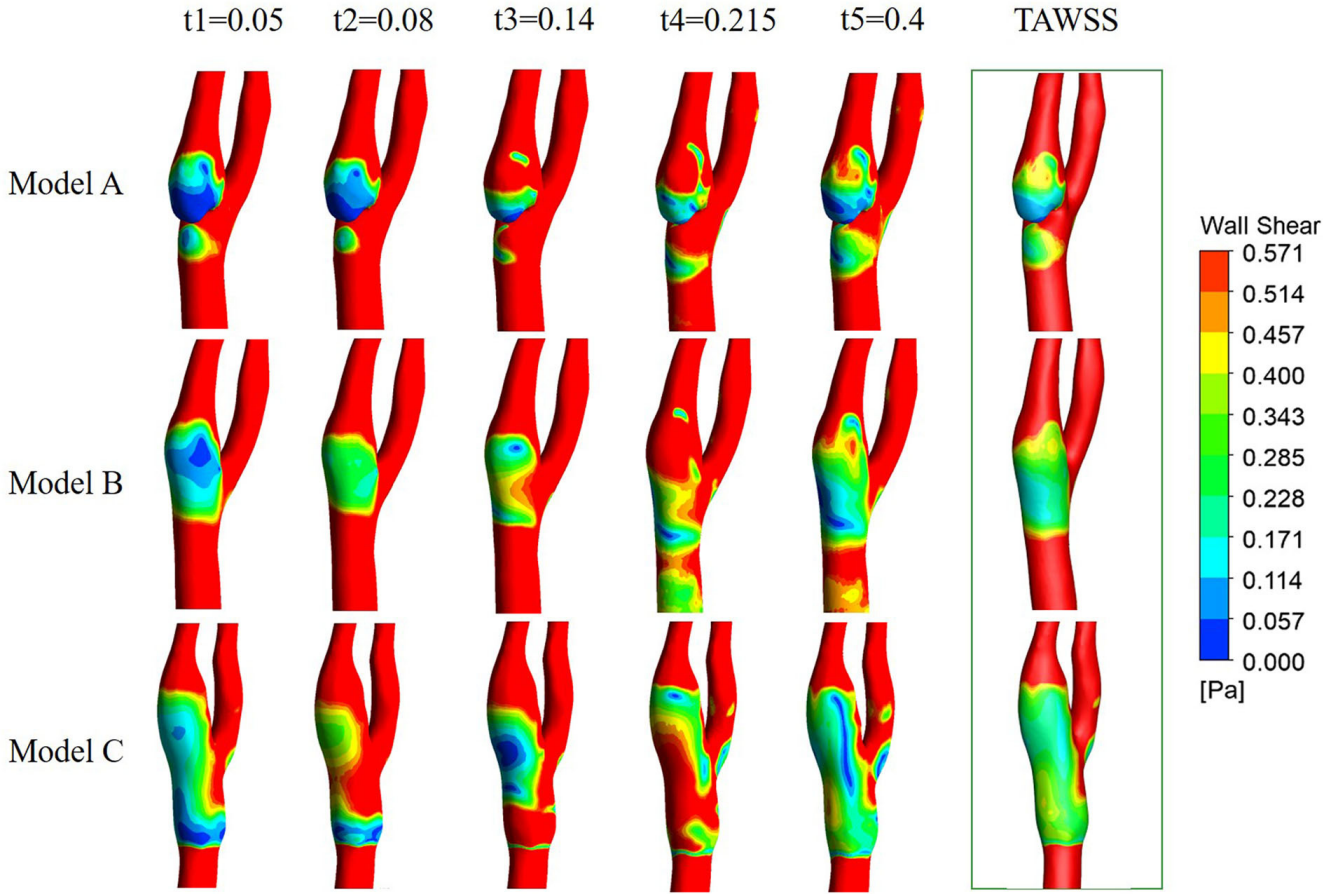
Contours of WSS at the five time points and the contours of TAWSS.

Figure 7 shows the WSS and TAWSS distribution contours of the three models. At the corner zone distal of the web in Model A, the wall shear stress is below 0.4 Pa throughout the cardiac cycle, and TAWSS was lower than 0.2 Pa, which is distinctly lower than in the other two models.

**Figure 8:**
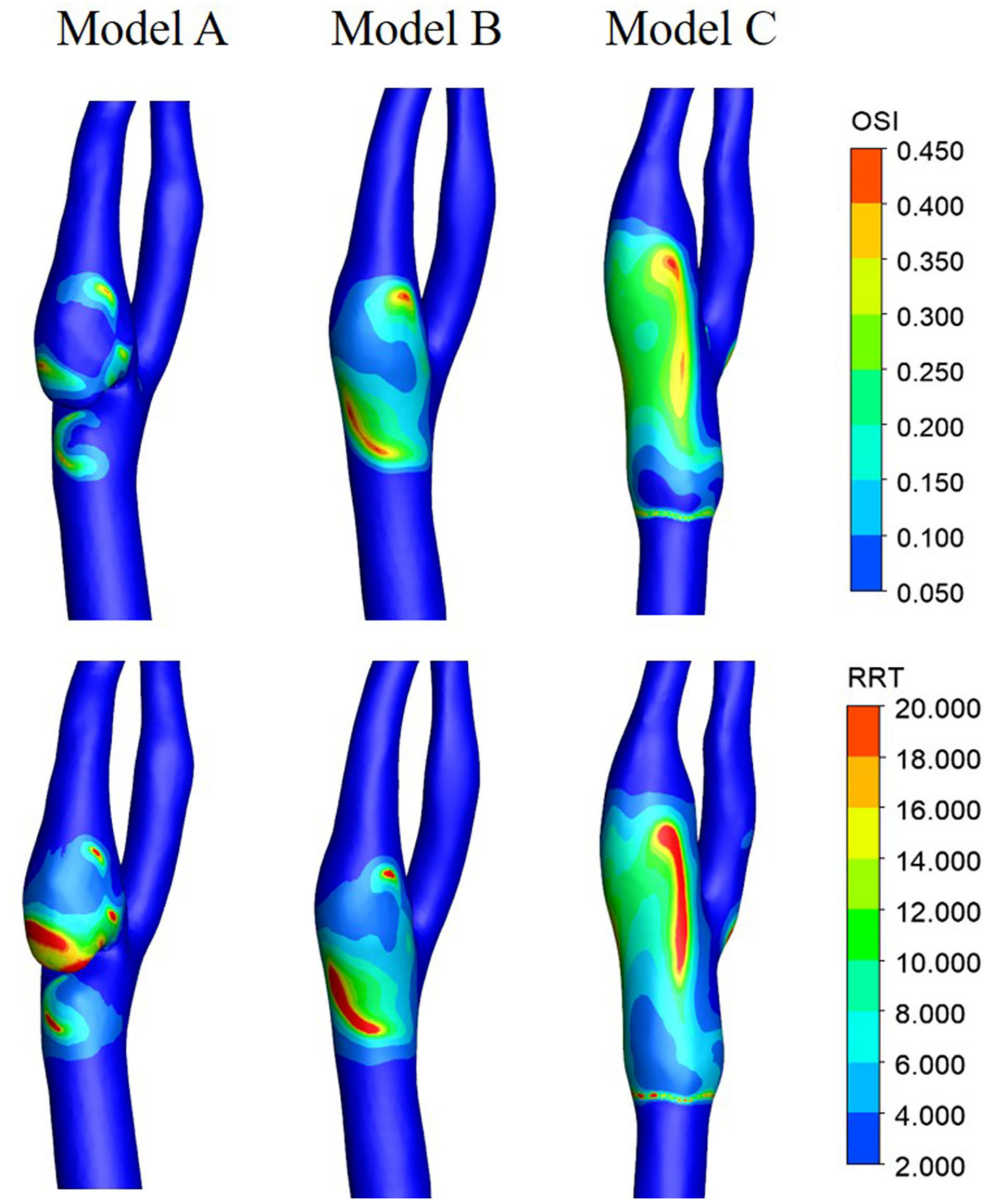
Contours of OSI and RRT.

As can be seen from Figure 8, relatively high OSI zones were observed in all three models, with relatively small zones in Model A and large zones in Model C. As the OSI represents the oscillation of WSS direction change, the OSI distribution features revealed that the WSS direction in Model A is relatively stable and in Model C is more unstable. This is probably because the stenting procedure increased the lumen diameter when oversizing the stent. The high values of RRT in Model A mainly concentrate on the corner zone distal to the web, while the distribution in Model B and Model C is relatively dispersed.

### The hemodynamics characteristics of the carotid siphon

Downstream of the carotid bifurcation, the blood flow in the three models remains stable along the internal carotid arteries until it reaches the carotid siphon.

**Figure 9:**
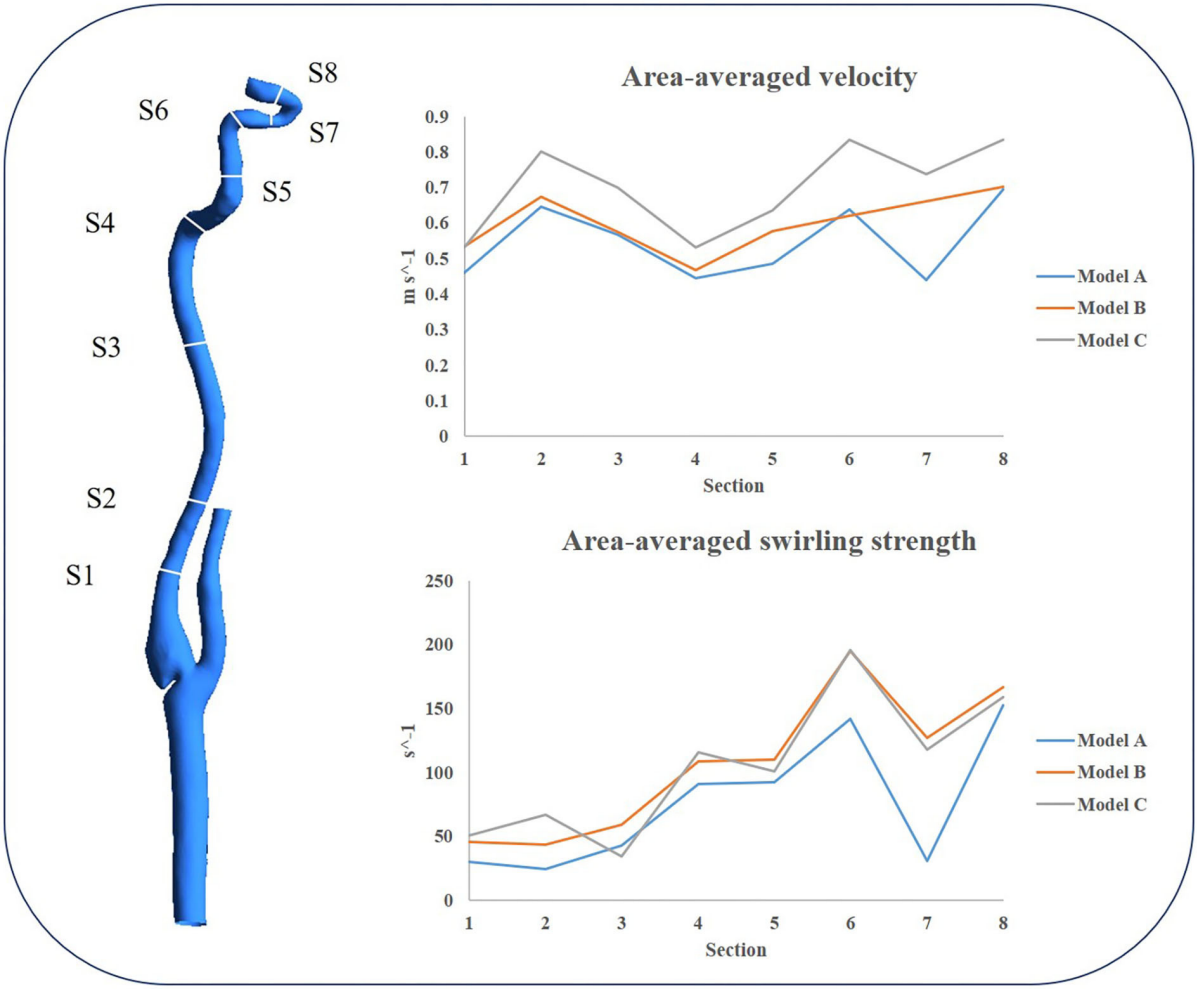
The cross sections are shown on the left; area-averaged velocity and area-averaged swirling strength in eight cross sections are shown on the right at the peak systole.

To quantitatively evaluate the effects of different treatments on the siphon area, eight cross sections were established (as shown on the left of Figure 9), and the velocity and swirling strength at the systolic peak time were shown on the right of Figure 9. Both the area-averaged velocity and the swirling flow strength in cross sections of model C were significantly higher than that of others, which would indicate greater kinetic energy and better washing of the inner wall of the carotid siphon.

**Figure 10:**
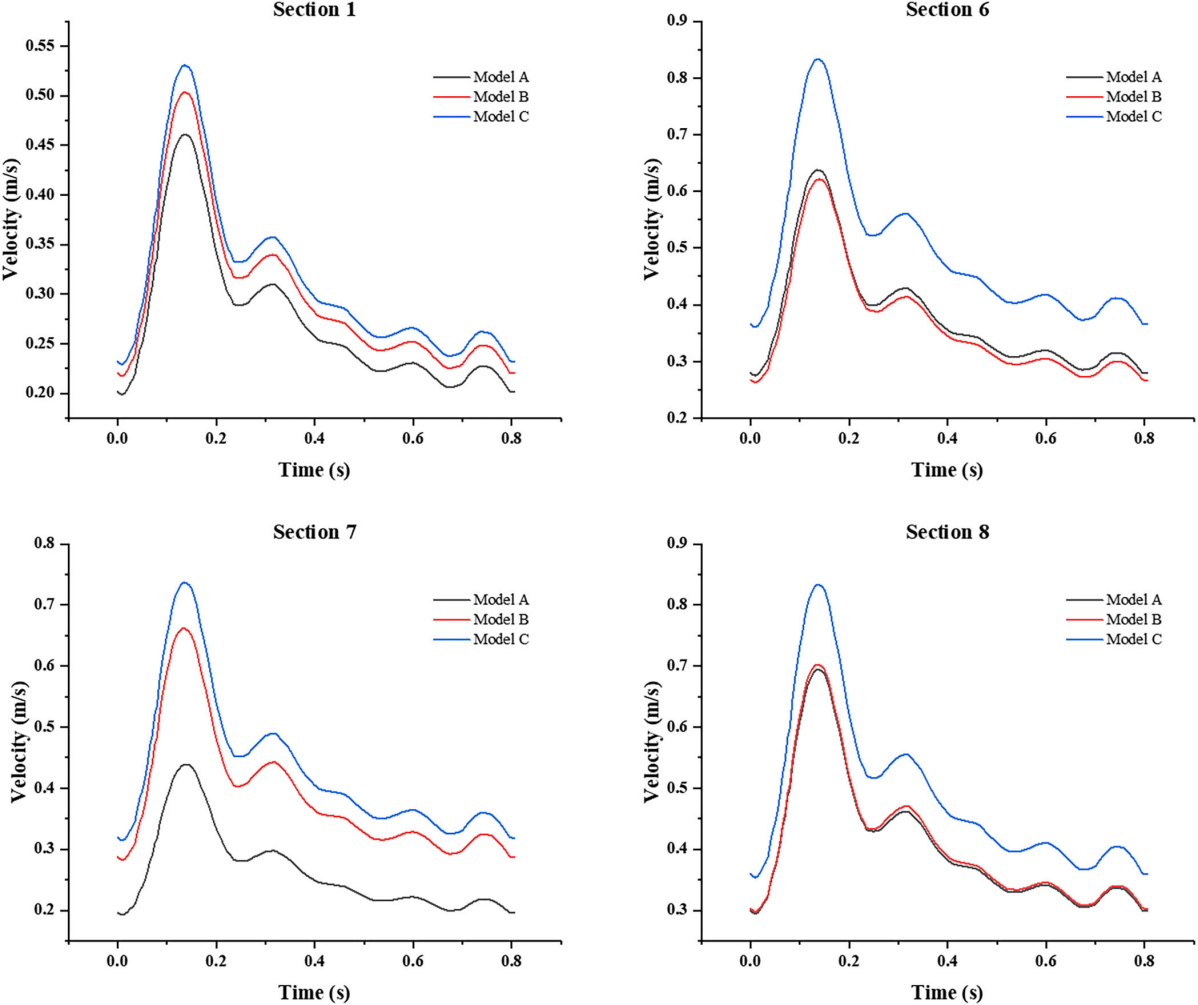
The change in area-averaged velocity in cross sections over time in one cycle.

**Figure 11:**
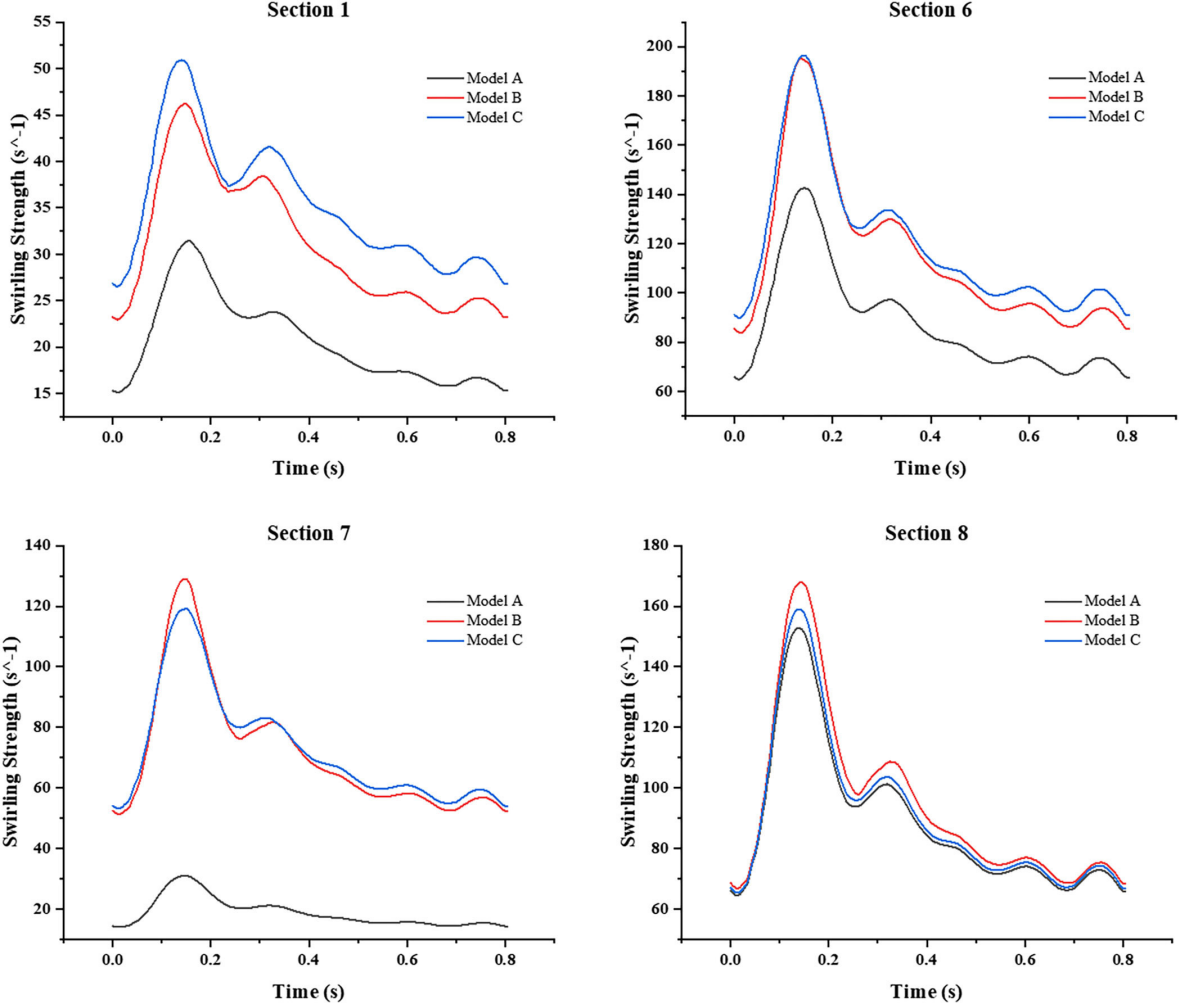
The change in area-averaged swirling strength in cross sections over time in one cycle.

In addition, we also focused on the changes of the area-averaged velocity and area-averaged swirling strength in cross sections of the three models over time in one cycle, as shown in Figures 10, 11. Compared with Model A, the average velocity of Model B and Model C increased, and the increase in Model C was more obvious. In addition, for the averaged-swirling strength, Model B and Model C have a similar degree of increase.

**Figure 12:**
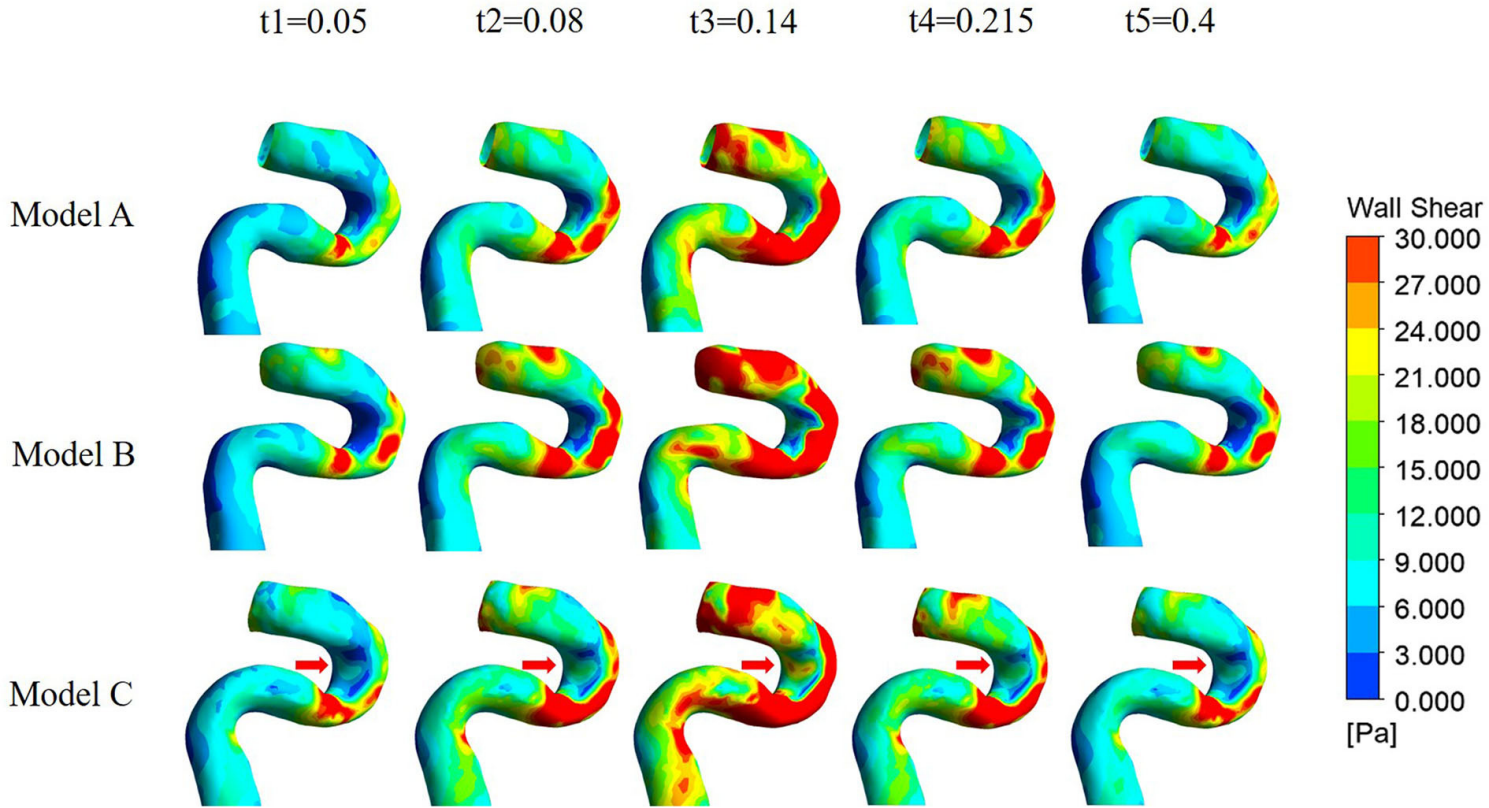
Contours of WSS of the three models at the carotid siphon.

The distributions of WSS at the carotid siphon in the three models are all uneven, being higher on the outer wall and lower on the inner wall. The difference between the three models appears on the inner side of the distal curve of the siphon (arrow in Figure 12). There was no significant difference between Model A and Model B, while Model C had the highest WSS.

Endothelial cell activation potential (ECAP) is considered to be an indicator of “thrombosis susceptibility”, which is defined as the ratio of OSI and TAWSS (31). As shown in Table 1, a larger ECAP means a lower risk of thrombosis. We calculated the area of higher OSI and lower TAWSS for three models, and the results showed that the risk of thrombosis was reduced in the model after both treatments. Table 2 shows the mean values of WSS, velocity, and swirling strength. Due to the presence of the web, the blood retention area is formed at the carotid bifurcation, and the scouring effect of blood flow on the vessel wall is weakened; this effect even extends to the siphon area. Both treatments can improve the condition to some extent, allowing an overall increase in WSS, velocity, and swirling strength, reducing the risk of thrombosis.

**Table 1:** Areas with an OSI >0.3, TAWSS < 0.1 Pa, and RRT >20 s in three models.

**Area(cm^2^)**	**OSI ≥ 0.3**	**TAWSS ≤ 0.1(Pa)**	**RRT ≥ 20(s)**
**Model**			
A	0.052	0.387	0.159
B	0.137	0.020	0.119
C	0.372	0.000	0.177

**Table 2:** Average values of WSS, velocity, and swirling strength for the three models.

**Average**	**WSS (Pa)**	**Velocity (m/s)**	**Swirling strength (s^−1^)**
**Model**			
A	7.404	0.410	51.330
B	7.812	0.449	58.770
C	8.638	0.449	53.210

## Discussion

Previous studies have speculated there is a significant association between the carotid web and recurrent ischemic strokes because of the disturbed flow pattern caused by the web tissue (2, 3, 32–36). The treatment strategy for the carotid web is now still under discussion, and the stenting treatment has been tried and encouraged for its minimally invasive advantage recently, while carotid endarterectomy has also been more widely used in clinics (2, 4, 11). This study then evaluated and compared how the two main treatments changed the hemodynamics at the carotid bifurcation area and the distal siphon area.

The results revealed that the carotid web can significantly influence the flow pattern and parameters at the carotid bifurcation, causing a recirculation zone distal to the web and vortex near the web orifice, accompanied by local lower velocity, lower WSS, higher Q vortex, and higher RRT, all of which have already been proven to have a close correlation with thrombosis and stroke *via* early platelet/clot accumulation and deposition (37–39). Although the OSI distal to the web was ‘unexpectedly' low behind the web, the velocity is also very low and the relative resident time is longer than others, which represents a very stable and still recirculation distal to the web and would definitely increase the risk of thrombosis formation.

The simulation results of Models B and C also revealed that both the endarterectomy and stenting treatments can narrow the recirculation region near the outer wall of the carotid bifurcation and sweep away the stable region formerly behind the web. However, among the two treatments, the stenting prolongs the low WSS area because of the enlargement of the lumen in the stenting oversizing procedure, which may increase the possibility of in-stent restenosis.

From the whole cardiac cycle, as the web tissue obstructs the flow through the carotid and then increases the flow resistance, there is lower area-averaged velocity and area-averaged swirling strength in the sections of the carotid web model and lower WSS on the inner side wall of siphon curve, which is more obvious at the peak systole. While in treatment models, the side effects of the web on the distal carotid siphon have been diminished, the area-averaged velocity and swirling strength of models B and C have been enhanced, especially in the stenting model. The oversized stent lumen decreased the blood flow resistance through the carotid bifurcation and resulted in higher swirling strength and WSS at the carotid siphon area, which may decrease the thrombosis risk there.

Finally, we integrated the parameters related to thrombosis in Tables 1, 2, and, from the mechanical point of view, either CEA or CAS can remove the negative effects of the web and play a therapeutic role. However, stenting can change the geometry of the carotid artery, resulting in changes in mechanical parameters that are different from those of the normal carotid artery, although our preliminary study cannot explain whether this change is actually beneficial from the clinical perspective.

To the best of our knowledge, this study was the first one to evaluate the two treatments for carotid web from the view of hemodynamics and also the first one to reveal how the carotid treatments influence the flow situation at the downstream siphon area. According to this study, both endarterectomy and stenting treatments could significantly diminish the side effects of the web and are feasible choices for web patients in hemodynamic views. The stenting treatment seems less feasible than endarterectomy because of the prolonged low WSS area and its high risk of in-stent restenosis. However, the other side of the coin is that stenting treatment is a minimally invasive surgery and would also decrease the flow resistance at the carotid bifurcation. Furthermore, more and more cutting-edge stent technologies are emerging and developing, such as 3D-printed patient-specific stents (40), more flexible stents, and bioabsorbable stents. Thus, more clinical trials are needed to evaluate the two treatments in the future.

## Limitations

One limitation of this study is that only one carotid web case was studied because it is clinically rare. Although this typical carotid bifurcation with a web can represent the common biomechanical features of the web and the treatments to a certain extent, caution is needed to interpret the results before collecting additional cases and conducting clinical validation.

Second, the fluid was treated as the laminar flow in the process of numerical calculation despite the presence of stenosis caused by the carotid web. Transitional effects of turbulence may occur in the stenotic carotid arteries with atherosclerotic plaques. Lee et al. have shown that the blood flow in the post-stenotic region is transitional or weakly turbulent during systole and laminar flow during diastole (41). Lancellotti et al. performed large-eddy simulations of stenotic carotid arteries to describe the transitional effects appearing in real vascular districts with realistic boundary conditions (42). However, the doppler anemometer (LDA) experiment results of Gijsen et al. have shown that flow instabilities might occur, but turbulence is not expected to occur under these conditions (43).

In addition, some studies considered fluid-structure interaction (FSI) modeling for stenotic carotids. Bennati et al. studied the carotids with different atherosclerotic plaques using FSI (44). Gao et al. performed stress analysis on four subjects with different plaque burdens by FSI simulations (45). Tang et al. developed the 3D FSI models and investigated the effect of inflammation on plaque stress/strain conditions (46). In this study, we assumed rigid walls in our computations, as did Domanin et al. (47). Previous studies have shown that this assumption may have some effect on the numerical values of the parameters, but the distribution characteristics are similar (48, 49). In addition, although fluid-structure interaction (FSI) is physiologically more plausible, whether the benefit in the accuracy of results is worth the additional computational burden is uncertain (50). Inspired by Gao et al. (51), gender differences in the carotid web may be of interest.

## Conclusion

The carotid web can significantly influence the flow environments in the carotid artery and increase the possibility of thrombosis stroke. Both endarterectomy and stenting treatments could significantly diminish the side effects of the web and are feasible choices for web patients in hemodynamic views. Besides, the treatments for the carotid web would also influence the flow patterns of distal carotid siphon arteries, especially for the stenting treatment. More innovational designs are needed to make the minimally invasive stenting treatment more beneficial, and more clinical trials are needed to further evaluate and compare the two treatments.

## Data availability statement

The original contributions presented in the study are included in the article/supplementary materials, further inquiries can be directed to the corresponding author/s.

## Author contributions

SR: Conceptualization, methodology, software, validation, formal analysis, data curation, writing—original draft, and visualization. QL: Conceptualization, methodology, validation, data curation, and writing-original draft preparation. ZC: Conceptualization, visualization, and investigation. XD: Supervision, validation, and writing—reviewing and editing. AS: Supervision, writing—reviewing and editing, project administration, and funding acquisition. JL: Supervision, writing— reviewing and editing, and project administration. All authors contributed to the article and approved the submitted version.

## Funding

This study was supported by the National Natural Science Foundation of China (Nos. 11872096, 12172033, and 32071311).

## Conflict of interest

The authors declare that the research was conducted in the absence of any commercial or financial relationships that could be construed as a potential conflict of interest.

## Publisher's note

All claims expressed in this article are solely those of the authors and do not necessarily represent those of their affiliated organizations, or those of the publisher, the editors and the reviewers. Any product that may be evaluated in this article, or claim that may be made by its manufacturer, is not guaranteed or endorsed by the publisher.
